# Shading Nets Modified Cluster-Zone Radiation, Bunch Sunburn Percentage and Berry Amino Acid Content in Cabernet Sauvignon: A Preliminary Study

**DOI:** 10.3390/plants15142183

**Published:** 2026-07-16

**Authors:** Gastón Gutiérrez-Gamboa, Miguel Araya-Alman, Sebastián Romero-Bravo, Marcos Carrasco-Benavides, Nicolás Verdugo-Vásquez, Manuel Chacón-Fuentes

**Affiliations:** 1 INIA Cauquenes, Instituto de Investigaciones Agropecuarias, Camino a Parral km 4, Cauquenes 3690000, Chile; 2 Centro de Investigación e Innovación VitiScience—CIA 250013, Facultad de Agronomía y Sistemas Naturales, Pontificia Universidad Católica, Santiago 7820436, Chile; nicolas.verdugo@inia.cl; 3 Centro de Desarrollo del Secano Interior, Departamento de Ciencias Forestales, Facultad de Ciencias Agrarias y Forestales, Universidad Católica del Maule, Talca 3460000, Chile; marayaa@ucm.cl; 4 Laboratorio de Agricultura Cuantitativa y Ecofisiología (LACE), Departamento de Ciencias Agrarias, Universidad Católica del Maule, km 6 Camino Los Niches, Curicó 3340000, Chile; sromero@ucm.cl (S.R.-B.); mcarrascob@ucm.cl (M.C.-B.); 5 INIA Raihuen, Instituto de Investigaciones Agropecuarias, Av. Esperanza s/n, Estación Villa Alegre, Villa Alegre 3650000, Chile; 6 Agriaquaculture Nutritional Genomic Center, CGNA, Temuco 4781158, Chile; manuel.chacon@cgna.cl

**Keywords:** amino acid, bioclimatic indices, fruit-zone microclimate, berry sunburn, photoselective nets

## Abstract

Shading nets are increasingly used in warm-climate vineyards to mitigate excessive cluster exposure. However, relatively few studies have evaluated their effects on fruit-zone microclimate and berry composition. This single-season field study evaluated the effects of conventional and photoselective shading nets in Cabernet Sauvignon on cluster-zone radiation, thermal conditions, bunch sunburn and powdery mildew percentage, berry maturity, and free amino acid content. Shading treatments significantly modified incident photosynthetically active radiation in the bunch zone, while air-temperature-derived thermal indices were not significantly affected. The conventional black Raschel net produced the greatest reduction in incident radiation, but this reduction was not associated with a decrease in sunburn or powdery mildew severity in bunches. The gray–pearl photoselective net maintained intermediate radiation levels and showed a lower severity of sunburn and powdery mildew, together with higher must pH and lower berry arginine content. The black–white photoselective net reduced berry soluble solids, while the conventional Raschel net increased berry histidine content, indicating that berry maturity and amino acid responses were treatment-dependent. These results provided preliminary field evidence on the effects of shading nets on Cabernet Sauvignon bunch sanitary and berry composition. Therefore, further multi-season and multi-site studies are needed before broader recommendations on shading-net use in warm-climate vineyards.

## 1. Introduction

In warm viticultural regions, excessive fruit-zone exposure combined with frequent heat events can elevate canopy and berry temperature beyond the optimal physiological thresholds, producing sunburn development and compromising berry composition and quality [1]. Under these conditions, berry sunburn becomes a relevant physiological disorder because it is driven by the combined effect of high radiation and temperature, affecting berry quality and reducing yield [2]. Therefore, viticultural practices aimed at moderating fruit-zone exposure should be evaluated not only by their effects on yield components, but also by their capacity to reduce the severity of bunch damage [3].

Shading nets have emerged as a practical viticultural strategy to mitigate excessive solar radiation in vineyards [1,3]. Conventional monofilament Raschel nets are commonly used as physical shade structures, mainly reducing the amount of radiation reaching the canopy and clusters [4]. In contrast, photoselective or light-dispersive nets, including pearl-type materials, are designed not only to reduce light intensity but also to modify the spectral composition and diffusion of incoming radiation to leaves [5,6]. These optical properties may alter the cluster microclimate and berry metabolism differently, making the comparison among net types relevant for cultivar-specific vineyard management [5,6].

Shading has been studied as a tool to delay ripening, reduce heat stress, and protect berry quality [7,8], but there is still limited field evidence on how different net types affect bunch sunburn in Cabernet Sauvignon. Previous studies have demonstrated that fruit-zone shading can reduce visual cluster damage and berry dehydration under high-radiation and heat-wave conditions [9]. However, the most published evidence has focused on yield losses, while fewer studies have quantified changes in bunch sunburn severity among different shading-net materials. This distinction is important because a treatment may not completely prevent the appearance of symptoms but may reduce the proportion of severely damaged berries [3]. Moreover, the effects of shading nets on berry primary metabolism, particularly nitrogenous compounds, remain less understood than their effects on sugars, acids, or phenolic compounds.

Amino acids are key berry metabolites from both physiological and enological perspectives as an important fraction of berry nitrogen that contributes to yeast nutrition during fermentation, acting as precursors of aroma-related compounds [10]. Arginine, glutamine, alanine, and proline are the main amino acids found in musts and are relevant for fermentation since proline is poorly assimilated by wine yeasts under the anaerobic conditions typical of alcoholic fermentation [11]. Modifications in the content of individual amino acids, the proline-to-arginine balance, and the pool of amino acid-derived aroma precursors in berries may have important implications for yeast nitrogen metabolism, fermentation kinetics, and the aromatic potential of the resulting wines [10,12]. Despite the fact that berry amino acid metabolism has been shown to respond to changes in cluster light exposure, most available published evidence comes from leaf removal, UV manipulation, or artificial light-exclusion approaches rather than from comparative evaluations of vineyard-scale shading-net technologies [13,14]. To the best of our knowledge, the effects of conventional Raschel nets and photoselective pearl nets on individual amino acid composition in Cabernet Sauvignon berries remain scarcely documented.

Shading nets may alter the cluster-zone microenvironment by modifying solar radiation transmission, light quality, light diffusion, berry surface temperature, air movement, and relative humidity around the bunches [1,3,15]. These changes may generate management-induced microenvironmental conditions within the vineyard, here interpreted as a cluster-zone microterroir, since terroir expression is strongly associated with the interaction among climate, soil, cultivar, and viticultural practices [16]. Therefore, meteorological and bioclimatic indicators, including growing degree days, cool night index, and the frequency of hours or days above critical temperature thresholds, are useful to characterize the local conditions experienced by berries under different shading treatments. Thermal risk indices are particularly relevant for evaluating shading net performance in warm-climate vineyards, where bunch sunburn is driven by the interaction between accumulated heat, high solar radiation, and short-term extreme temperature events. Threshold-based risk indices, including hours or days with temperatures above 30 or 35 °C, provide a more targeted assessment of heat stress episodes associated with berry overheating, dehydration, and visible sunburn symptoms [17]. These conditions are also relevant for disease-related variables, particularly powdery mildew, whose development can be affected by sunlight exposure, UV radiation, tissue temperature, and cluster-zone ventilation [18].

Therefore, this study evaluated the effects of conventional and photoselective shading nets on cluster-zone radiation, thermal conditions, bunch sunburn and powdery mildew percentage, technical maturity, and berry amino acid content in Cabernet Sauvignon during one growing season.

## 2. Results

### 2.1. Cluster-Zone Incident Radiation and Thermal Accumulation

Table 1 shows the cluster-zone incident radiation, meteorological, bioclimatic, and thermal risk indices. Shading net statistically affects photosynthetically active radiation, whereas meteorological, bioclimatic, and thermal risk indices did not differ between non-covered, defoliated, and shaded vines. Basal leaf removal provided the most exposed cluster-zone environment, reaching the highest radiation incidence, followed by the non-covered vines. The conventional Raschel shading nets showed the strongest shading effect around the clusters, reducing photosynthetically active radiation in 61.7 and 54.2% of basal defoliation and uncovered vines, respectively. This reduction was greater than that observed by the vines covered using photoselective shading nets.

### 2.2. Vine Water Status and Leaf Chlorophyll Index

**Figure 1:**
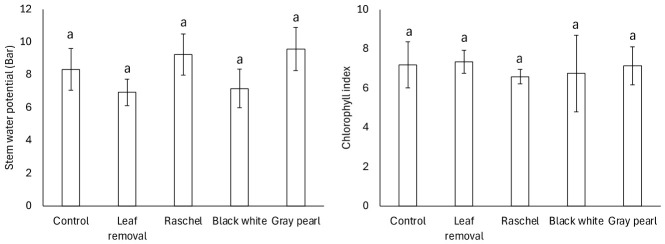
Stem water potential (**left**) and leaf chlorophyll index (**right**) of Cabernet Sauvignon vines subjected to basal leaf removal, shading nets, and control (uncovered vines). Shading treatments included conventional (Raschel net: 35% of shading) and photoselective (black–white and gray–pearl nets: 16% and 22% of shading, respectively). Different letters within each row indicate significant differences between treatments according to Duncan’s multiple range test (*p*-value ≤ 0.05).

Figure 1 shows vine stem water potential and leaf chlorophyll index. These variables showed no significant differences among the treatments. Stem water potential ranged between −6.9 and −9.6 bar in vines subjected to basal leaf removal and vines covered with gray–pearl photoselective nets, respectively, whereas leaf chlorophyll index ranged from 6.6 to 7.3 in leaves from vines covered with conventional nets and vines subjected to basal leaf removal, respectively.

### 2.3. Yield Components, Berry Maturity, Bunch Sunburn, and Sanitary Status at Harvest

Table 2 shows yield components, berry maturity parameters, bunch sunburn, and powdery mildew variables determined at harvest. Yield per vine, bunch weight, and berry weight did not differ significantly among treatments. The number of bunches per vine was the only yield component affected by the treatments, with basal leaf removal showing a higher number of bunches than black–white-covered and uncovered vines. Soluble solids content was the lowest in berries from vines covered with the black–white net (20.6 °Brix).

Must pH has differed significantly among treatments. Musts from vines covered with Raschel nets had the lowest pH, whereas musts from vines covered with gray–pearl photoselective nets had the highest pH. Bunch sunburn incidence did not differ significantly among treatments and ranged from 76.7 to 86.5%, whereas bunch sunburn severity differed significantly, ranging from 11.6 to 20.1% in bunches from vines covered with gray–pearl and conventional shading. Similarly, powdery mildew severity on bunches varied significantly among treatments, with values ranging from 7.3 to 21.0%. The conventional Raschel nets showed significantly higher disease severity than basal leaf removal and photoselective shading nets, although values were not significantly different from those observed in uncovered vines. Powdery mildew incidence differed significantly among treatments, ranging from 62.63 to 86.81%. The conventional Raschel nets increased the incidence of this disease significantly compared to uncovered control vines and gray–pearl photoselective net treatments, but did not differ significantly from basal leaf removal and the black–white photoselective nets.

### 2.4. Amino Acids in Berries at Harvest

Table 3 shows the berry amino acid content and amino acid-derived indices. The content of most of the individual amino acids did not differ significantly among treatments. Arginine content differed significantly among treatments, ranging from 1.40 to 3.68 mg N kg^−1^ in berries from vines covered with gray–pearl photoselective nets and basal leaf removal, respectively. Berries from gray–pearl-covered vines had significantly lower arginine content than the berries from vines subjected to basal leaf removal and covered with conventional Raschel and black–white shading nets. Histidine content in berries was significantly lower in basal leaf removal than in the berries collected from vines covered with conventional shading nets (1.62 and 4.56 mg N kg^−1^, respectively). The proline-to-arginine ratio, total amino acid content, total amino acid content excluding proline, and aromatic precursor nitrogen content did not differ significantly among treatments.

### 2.5. Exploratory Correlation and Multivariate Analysis

Supplementary Figure S1 shows the exploratory correlation analysis among the studied variables. Bunch sunburn severity was positively correlated to powdery mildew severity (r = 0.94) and arginine content in berries (r = 0.91), while negative correlations were observed with must pH (r = −0.91), minimum temperature (r = −0.95), and mean temperature (r = −0.90). The percentage of bunch sunburn incidence was negatively correlated with the cool night index (r = −0.90). The percentage of powdery mildew severity was negatively correlated with must pH (r = −0.90) and mean temperature (r = −0.95). Mean temperature was positively correlated with the proline-to-arginine ratio (r = 0.95) and aromatic precursor nitrogen (r = 0.89). Among thermal variables, maximum temperature was positively correlated to the number of days above 35 °C (r = 0.91), the hours above 30 °C (r = 0.88), and the hours above 35 °C (r = 0.96). Arginine content in berries was negatively correlated with glutamic acid content (r = −0.94) and total amino acid content excluding proline (r = −0.95).

**Figure 2:**
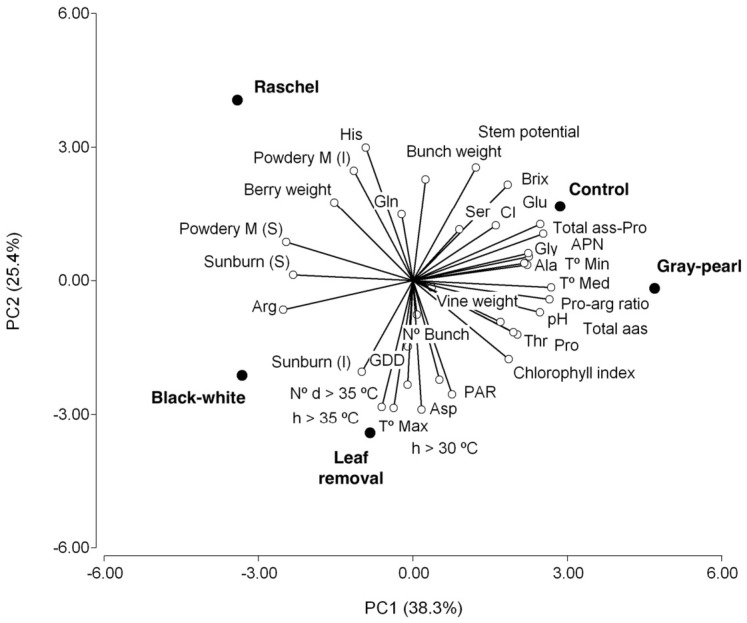
Exploratory principal component analysis of agronomic, physiological, microclimatic, sanitary, and berry compositional variables in Cabernet Sauvignon vines subjected to basal leaf removal, shading-net treatments, and uncovered control vines. Abbreviations: (S): severity; (I): incidence; M: mildew; N°: number; Stem potential: stem water potential; PAR: photosynthetically active radiation; T° Min: minimum temperature; T° Max: maximum temperature; T° Med: mean temperature; GDD: growing degree days; CI: cool night index; h > 30 °C: number of hours above 30 °C; h > 35 °C: number of hours above 35 °C; N° d > 35 °C: number of days with temperature above 35 °C; Gly: glycine; Ala: alanine; Pro: proline; Arg: arginine; Gln: glutamine; Ser: serine; Asp: aspartic acid; Glu: glutamic acid; Thr: threonine; His: histidine; Pro-Arg ratio: proline-to-arginine ratio; Total AAs: total amino acids; Total AAs-Pro: total amino acids excluding proline; APN: aromatic precursor nitrogen.

Figure 2 shows the exploratory principal component analysis (PCA) performed using yield components, berry maturity, bunch sunburn and powdery mildew, meteorological and thermal risk indices, vine physiological variables, and berry amino acid composition. The first two principal components explained 63.7% of the total variance, with PC1 accounting for 38.3% and PC2 for 25.4%. The third component explained an additional 22.4%, increasing the cumulative explained variance to 86.1%, while the fourth component completed the remaining variability. The cophenetic correlation was 0.869, indicating an adequate representation of the multivariate structure.

PC1 was mainly associated with berry compositional and canopy thermal variables. Positive correlations with PC1 were observed for the proline-to-arginine ratio (r = 0.97), mean temperature (r = 0.98), total amino acid content excluding proline (r = 0.93), glutamic acid content (r = 0.91), must pH (r = 0.90), minimum temperature (r = 0.82), aromatic precursor nitrogen (APN) content (r = 0.82), glycine content (r = 0.81), and alanine content (r = 0.79). In the opposite direction, PC1 was negatively correlated with arginine content (r = −0.92), percentage of powdery mildew severity (r = −0.90), percentage of sunburn severity (r = −0.85), and berry weight (r = −0.56). The uncovered vines and vines covered with gray–pearl photoselective nets were positioned toward the positive side of PC1, whereas the conventional Raschel net and the black–white photoselective net were located toward the negative side of the component.

PC2 separated treatments mainly according to heat risk, radiation, and some physiological and compositional variables. Positive correlations with PC2 were observed for histidine content (r = 0.89), stem water potential (r = 0.76), percentage of powdery mildew incidence (r = 0.74), bunch weight (r = 0.68), soluble solids content (r = 0.64), and berry weight (r = 0.52). Negative correlations with PC2 were observed for hours above 30 °C (r = −0.86), maximum temperature (r = −0.85), hours above 35 °C (r = −0.84), aspartic acid content (r = −0.76), number of days above 35 °C (r = −0.69), PAR (r = −0.66), and percentage of sunburn incidence (r = −0.61). The vines subjected to basal leaf removal were positioned toward the negative side of PC2, close to thermal risk variables and PAR, whereas conventional Raschel nets were positioned toward the positive side of PC2, in the direction of histidine content, percentage powdery mildew incidence, stem water potential, and berry weight.

## 3. Discussion

The present single-season field study provided preliminary evidence that conventional and photoselective shading nets modified the cluster-zone environment mainly through changes in incident photosynthetically active radiation, while air-temperature-derived meteorological, bioclimatic, and thermal risk indices were not significantly affected [2,19]. In this trial, Cabernet Sauvignon rows were oriented approximately to a north–south axis, with an azimuth of 22° from north. Row orientation may also have influenced the distribution of cluster-zone incident radiation [20]. The conventional Raschel shading net produced the strongest attenuation of incident photosynthetically active radiation, in agreement with its higher nominal shading factor compared to photoselective nets. This reduction in incident photosynthetically active radiation was not reflected in lower bunch sunburn and powdery mildew severity [3,21]. The greater shading effect induced by the conventional Raschel net cannot be attributed only to the reduction in incident PAR. This response supports the interpretation that shading nets may act as microclimate-modifying materials whose effects could depend on the combined influence of radiation attenuation, material optical properties, canopy exposure, and local bunch-zone conditions [1,22]. Despite the fact that variables such as airflow, relative humidity, UV transmission, berry surface temperature, and radiative exchange were not directly measured in the present study, they may have contributed to the observed differences in this field trial [21]. In addition, plastic shading nets have specific absorptivity, reflectance, transmittance, and emissivity properties [22], meaning that conventional nets may modify the local radiative balance during afternoon exposure despite reducing incident radiation at this time. This physical mechanism is particularly relevant because row orientation modulates the temporal distribution of direct and diffuse radiation within the canopy, thereby influencing the timing and intensity of the radiative load received by each canopy side and the bunch zone [23]. The afternoon exposure observed on the western side of the canopy may have intensified the interaction among incoming radiation, net material properties, and the fruit-zone conditions, potentially limiting heat dissipation and favoring a more humid bunch microenvironment.

The contrasting behavior of the shading materials suggests that their effect depended on the fruit-zone microenvironment generated by each net. Despite the fact that the conventional nets produced the strongest reduction in cluster-zone radiation, this treatment was also associated with a high percentage of bunch sunburn severity and the highest percentage of powdery mildew incidence and severity. In contrast, photoselective gray–pearl nets maintained intermediate radiation levels, but they showed the lowest percentage of sunburn and powdery mildew severity. This divergence indicates that excessive radiation attenuation did not necessarily improve bunch condition. Instead, the response under conventional nets may reflect a less favorable balance among radiation filtering, UV reduction, air renewal, relative humidity, and local heat dissipation around the clusters [22]. The interpretation of sunburn responses should also consider that the thermal characterization in this study was based on air temperature recorded within the cluster zone, whereas berry surface temperature was not directly measured. This distinction is relevant because sunburn development is more closely associated with the thermal load experienced by the berry surface than with surrounding air temperature alone [2]. Berry surface temperature may differ from cluster-zone air temperature depending on incident radiation, berry exposure, canopy side, air movement, and the optical and thermal properties of the shading material [2]. Despite the fact that the air-temperature-derived indices provided useful information on the thermal conditions within the fruit zone, they cannot be used to directly infer berry overheating. On the other hand, reduced sunlight and radiation exposure can favor cryptogamic disease development in grapevine clusters. Austin and Wilcox [24] used artificial shading and spectral-filtering experiments in vineyards to separate the effects of radiation and tissue heating on grapevine powdery mildew. By filtering UV radiation from sunlight reaching the vines, they reported a 20–40% increase in cluster disease severity relative to the exposed control. In the present study, the positive association between the percentage of bunch sunburn and powdery mildew severity suggests that both responses may have been favored by a common bunch-zone condition involving restricted air renewal, altered radiative exchange, and higher local humidity. The importance of this interaction may vary with the seasonal climatic conditions and disease pressure during ripening [25].

The maturity response observed in this study indicates that nominal shading percentage was not sufficient to explain the changes in berry soluble solids and must pH. This is relevant because photoselective nets combine radiation attenuation, spectral modification, and light diffusion, creating optical conditions in the cluster zone that may influence berry metabolism differently from conventional shading nets [26]. In Cabernet Sauvignon, previous studies have shown that fruit-zone shading can delay sugar accumulation and modify organic acid degradation, especially under intense or prolonged radiation exclusion [9,15]. However, the studies using colored nets or photoselective films have also reported that berry composition may change even when moderate levels of radiation are transmitted, because these materials alter the balance between direct radiation, diffuse light, heat load, and berry exposure [5,6]. This interpretation is further supported by the contrasting berry-composition responses observed in the present study, particularly the reduction in the content of soluble solids in berries under the black–white net despite its lower nominal shading factor, and the divergent effects of conventional and photoselective nets on must pH. The contrasting responses of berry soluble solids and must pH suggest that each net modified the cluster-zone optical and thermal environment differently, affecting sugar accumulation and organic acid balance in a material-specific manner.

The amino acid response showed a compound-specific pattern, as significant treatment effects were observed for arginine and histidine content in berries, while the overall amino acid pool and derived indices were not significantly modified. The decrease in berry arginine content under gray–pearl photoselective nets is relevant from a berry-composition perspective because arginine is one of the main free amino acids found in berries. However, in the absence of broader changes in the amino acid pool, this response should be interpreted as compound-specific rather than as evidence of a generalized shift in berry nitrogen composition [10]. The exploratory correlation analysis also showed a negative association between arginine and glutamic acid content in berries. This relationship is physiologically plausible because glutamate is a central amino donor in nitrogen metabolism and participates in the biosynthetic network leading to arginine formation [12]. Thus, the opposite behavior between arginine and glutamic acid may reflect a redistribution of nitrogen among related amino acid pools rather than a generalized increase or decrease in berry amino acid content [12]. The regulation of berry amino acids under different light environments appears to be compound-specific. The studies in viticulture about shading nets have shown that changes in fruit-zone exposure can modify amino acid accumulation according to cultivar, berry tissue, developmental stage, and the metabolic role of each amino acid [14].

These results provide preliminary guidelines for the use of shading nets as cultivar- and site-specific adaptation tools in vineyards located in warm climates. To our knowledge, this is one of the first field studies to integrate conventional and photoselective shading nets with cluster-zone meteorological, bioclimatic, and thermal risk indices, together with sunburn severity, powdery mildew, and berry amino acid composition. These indices provide a broader framework for interpreting the effects of shading materials, as they account not only for changes in incident radiation but also for cumulative thermal conditions and the frequency of heat-risk thresholds experienced by bunches. Nevertheless, a limitation of the present study is that the berry surface temperature was not directly recorded. This variable is particularly important because it represents the thermal condition most directly experienced by the berry epidermis and is therefore more closely linked to sunburn development than air temperature alone [2]. In addition, the amino acid results provided early evidence that photoselective nets may affect the content of specific amino acids in berries, an aspect that remains scarcely documented in vineyard-scale shading studies. Future studies should evaluate these materials over several seasons and in cultivars with different phenology behavior, including early- and late-ripening varieties, to determine whether their effectiveness is stable across climatic conditions. Seasonal measurements of berry surface temperature, net surface temperature, UV and near-infrared transmission, wind speed, relative humidity, and vapor pressure deficit at the bunch level, separated by canopy side and time of day, would also be necessary to clarify the mechanisms underlying the responses observed in this field trial. These measurements would be necessary to confirm whether the hypothesized effects on air renewal, humidity, heat dissipation, and UV exposure explain the responses observed in this single-season field trial.

## 4. Materials and Methods

### 4.1. Study Site and Plant Material

The experiment was conducted during the 2024–2025 growing season in a Cabernet Sauvignon vineyard located at the Escuela Agrícola Superior de Molina, Quechereguas sector, Molina, Maule Region, Chile (35°05′52″ S, 71°16′24″ W; WGS 84). The site is located in the Maule Valley, a viticultural area characterized by winter rainfall and dry, warm summers. The vineyard was planted in 1998 and grafted in 2015 using massal-selection Cabernet Sauvignon plant material. The vines were trained to a vertical shoot-positioned system, spaced at 2.3 m × 1.3 m, and oriented to a north–south axis, with an azimuth of 22° from north. The vineyard was managed under drip irrigation using emitters with a discharge rate of 4 L h^−1^. Irrigation was scheduled according to plant water status and applied when the average midday stem water potential (Ψstem) approached −1.2 MPa. Standard local viticultural practices were applied uniformly across all treatments. The soil belongs to the Lontué series, a recent alluvial soil with sandy-loam texture, rapid permeability, and excessive drainage.

### 4.2. Experimental Design and Arrangement of Treatments

**Figure 3:**
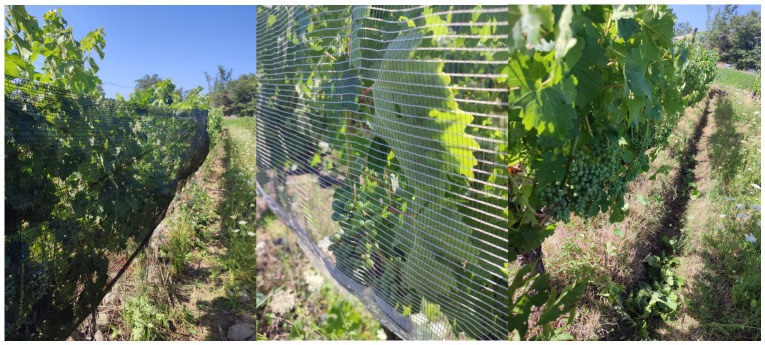
Shading-net treatments and basal leaf removal applied to grapevines: conventional Raschel netting on the canopy and cluster zone (**left**), photoselective black–white netting on the canopy and cluster zone (**middle**), and basal leaf removal exposing bunches to natural radiation conditions (**right**).

The experiment was arranged as a completely randomized design with five treatments and three replicates per treatment (Supplementary Figure S2). The treatments were established in vineyard plots arranged across three rows, with one untreated row maintained between adjacent treatment plots to reduce interference among treatments. In the shaded treatments, the nets were installed over the fruiting zone of the assigned plots, covering the bunch zone and the whole canopy (Figure 3). At least five vines were maintained at the ends of each plot to minimize border effects and potential interference between adjacent treatments, and measurements were restricted to central vines within each replicate. The nets were attached to the trellis posts and fixed along the vertical shoot-positioned canopy using plastic ties and manual staplers. The conventional and photoselective nets were placed on the western side of the rows.

The treatments were established at the pea-size berry stage, corresponding approximately to BBCH 75 in the modified Eichhorn–Lorenz phenological scale, when berries reached pea-sized, and bunches began to hang [27]. The trial consisted of five treatments: (i) uncovered control vines; (ii) basal leaf removal; (iii) conventional Raschel net; (iv) black–white photoselective net; and (v) gray–pearl photoselective net. (i) The uncovered control consisted of vines without basal leaf removal and without the installation of shading nets. (ii) Basal leaf removal consisted of manually removing the six basal leaves from the fruiting zone to increase cluster exposure. (iii) The conventional shading treatment consisted of a black Raschel net with 35% nominal shading. The manufacturer indicates that this net had approximately 1.2 to 1.4 threads cm^−2^ in the weft, equivalent to 3 threads per inch, and approximately 2.6 meshes per cm. Two photoselective shading treatments were evaluated. (iv) The black–white photoselective net had 16% of nominal shading and a thread density of 2.6 × 3.0 threads cm^−2^, whereas the gray–pearl photoselective net had 22% of nominal shading and a thread density of 4 × 4 threads cm^−2^, according to manufacturer specifications. At each shading-net treatment, net sections were cut to approximately 7 m in length and 1 m in width and installed along the fruit and canopy zone between two consecutive trellis posts (Figure 3).

### 4.3. Cluster-Zone Temperature and Incident PAR Monitoring and Calculation of Meteorological, Bioclimatic, and Thermal Risk Indices

Cluster-zone temperature was monitored independently in each field trial replicate using high-resolution iButton temperature data loggers (Thermochron DS1921G-F5, Analog Devices, Wilmington, MA, USA). The self-contained temperature sensors, with an operating range of −40 to 85 °C, were programmed to record data at hourly intervals throughout the experimental period from pea-size berry stage and continuing through harvest. Sensors were installed in the central part of each replicate, close to a representative bunch within the fruiting zone. Each sensor was mounted using an iButton snap-in fob (DS9093F, Analog Devices, USA) and placed inside a ventilated, perforated radiation shield to reduce direct solar exposure while allowing air exchange around the sensor. After harvest, temperature data were downloaded using a USB 2.0 Full-Speed iButton adapter (DS9490B, Analog Devices, Wilmington, MA, USA) and the corresponding iButton data acquisition software.

Raw hourly temperature data were checked for consistency before calculating daily minimum, maximum, and mean temperature for each replicate. These records were then used to calculate cluster-zone meteorological, bioclimatic, and thermal risk indices following the approach described by Verdugo-Vásquez et al. [17,28], with adaptations to the scale and duration of the present experiment. Meteorological indices included minimum, maximum, and mean temperature. Bioclimatic indices included growing degree days, calculated using a 10 °C base temperature, and the cool night index [29,30]. Thermal risk indices were calculated as threshold-based indicators, including the number of hours with temperatures above 30 °C and 35 °C, and the number of days with afternoon temperatures above 35 °C [17]. These indices were calculated for each replicate from the date of net installation to harvest in order to describe the thermal conditions imposed by each fruit-zone treatment.

Intercepted photosynthetically active radiation (PARi) was determined one week after net installation using an AccuPAR LP-80 ceptometer (METER Group, Pullman, WA, USA). Measurements were taken in the lower part of the canopy, within the fruiting zone, where the shading nets were expected to modify the light environment. For each replicate, the ceptometer probe was positioned horizontally beneath the canopy and close to the bunch zone, avoiding direct contact with leaves, shoots, or clusters. Readings were taken under stable light conditions and used to characterize the amount of PAR intercepted by the canopy and shading treatments.

### 4.4. Stem Water Potential and Chlorophyll Index

Stem water potential (Ψstem) was measured at midday using a pressure chamber (model 600, PMS Instrument Co., Corvallis, OR, USA) according to the methodology exposed by Acevedo-Opazo et al. [31]. Measurements were performed between 12:00 and 14:00 h on clear days. In each replicate, fully expanded and healthy leaves located close to the fruiting zone were selected. The leaves were enclosed in plastic bags and aluminum foil for at least 2 h before measurement to suppress transpiration and allow equilibration between leaf water potential and stem xylem water potential. After equilibration, leaves were excised and immediately measured in the pressure chamber and the values were expressed in bar. Leaf chlorophyll status was assessed non-destructively using a SPAD chlorophyll meter (SPAD-502Plus, Konica Minolta, Osaka, Japan). Measurements were taken on fully expanded, healthy leaves from the same canopy zone used for stem water potential assessment. The sensor was placed on the central portion of the leaf blade, avoiding the midrib and major veins. Five readings were taken per replicate and averaged to obtain one representative SPAD chlorophyll index value.

### 4.5. Yield Components, Berry Maturity, Bunch Sunburn, and Powdery Mildew Assessment

Bunches from the vines included in each replicate were collected and weighed in the field at harvest. The yield per vine was calculated by dividing the total harvested fruit weight of each replicate by the number of evaluated vines and was expressed as kg per vine. The number of bunches per vine was recorded by counting all harvested bunches by replicate. A representative berry sample was collected from each replicate at harvest to determine berry maturity parameters. Berry weight was determined from a subsample of fifty berries. The berries previously harvested were crushed, and the must was used to determine the content of soluble solids and pH. Soluble solids were measured using a digital refractometer and expressed as °Brix, while must pH was determined using a calibrated pH meter.

Bunch sunburn was visually assessed at harvest following an adapted berry-level classification based on sunburn damage type and intensity published by Gambetta et al. [32]. Bunch sunburn incidence was calculated as the percentage of bunches showing visible sunburn symptoms. Bunch sunburn severity was visually estimated as the percentage of the bunch affected by sunburn symptoms, including browning and necrosis, and expressed as a percentage of the affected bunch area. Powdery mildew incidence was calculated as the percentage of bunches showing visible symptoms [33]. Powdery mildew severity was estimated visually as the percentage of bunch surface area affected by powdery mildew symptoms [33]. Bunch sunburn and powdery mildew assessments were performed after harvest on all bunches collected from the evaluated vines within each replicate. The number of bunches assessed ranged from 67 to 81 per replicate, resulting in a total of 379 evaluated bunches. Two trained observers with previous experience in the assessment of grapevine bunch disorders and disease symptoms jointly conducted the visual evaluations. Before scoring, observers used the same visual criteria and reference descriptions for sunburn damage and powdery mildew symptoms in order to harmonize the evaluation procedure. Incidence and severity values were averaged for each experimental unit and expressed as percentages. Bunch-level observations were averaged by replicate to obtain one replicate-level value before statistical analysis.

### 4.6. Amino Acid Determination in Berries

Free amino acids in Cabernet Sauvignon berries were determined using a reversed-phase HPLC-DAD method after pre-column derivatization with 9-fluorenylmethyl chloroformate (Fmoc-Cl), adapted from the method described by Kahsay et al. [34]. This method was selected because Fmoc-Cl reacts with both primary and secondary amino acids under mild alkaline conditions, allowing the simultaneous determination of free amino acids, including proline. Berry samples by replicate were collected at harvest, freeze-dried and ground to obtain a homogeneous powder. Briefly, 0.5 g of lyophilized berry material was weighed and extracted with 5 mL of 0.1 N HCl. The mixture was sonicated for 30 min at 37 kHz, maintaining the extraction temperature between 20 and 30 °C. Extracts were then centrifuged at 4000 rpm for 10 min, and the supernatant was filtered through a 0.22 μm hydrophilic PVDF membrane filter. Before derivatization, the filtered extract was diluted five-fold with the extraction solvent.

For derivatization, 100 μL of each sample extract or standard solution was transferred to a 2 mL Eppendorf tube. Then, 200 μL of 0.5 M sodium borate buffer at pH 8.6 and 400 μL of 6 mM Fmoc-Cl prepared in acetonitrile were added. The mixture was vortexed and incubated at room temperature for 10 min to allow complete derivatization. The reaction was stopped by adding 300 μL of 12.5 mM 1-adamantylamine prepared in water:acetonitrile. The mixture was incubated for an additional 2 min and centrifuged at 10,000× *g* for 5 min. The supernatant was transferred to HPLC vials for chromatographic analysis. The chromatographic separation was performed by HPLC-DAD using a Poroshell 120 EC-C18 column (3.0 × 100 mm, 2.7 μm; Agilent Technologies, Santa Clara, CA, USA). Mobile phase A consisted of 50 mM sodium acetate buffer adjusted to pH 4.15, and mobile phase B consisted of acetonitrile. The flow rate was 0.7 mL min^−1^, the column temperature was maintained at 25 °C, and the injection volume was 10 μL. Separation was carried out under gradient elution from 15 to 40% mobile phase B, followed by re-equilibration to the initial conditions.

Amino acids were identified by comparison with external standards and quantified using calibration curves prepared from a commercial amino acid standard mixture. Standard solutions were derivatized using the same procedure as the samples. The final calibration range after derivatization was 2.5–80 μM. Amino acid concentrations were expressed as mg N kg^−1^ of berry fresh weight, considering the nitrogen contribution of each amino acid.

The proline-to-arginine ratio was calculated by dividing the concentration of L-proline by that of L-arginine. Total amino acids were calculated as the sum of all quantified free amino acids. Total amino acids excluding proline were calculated by subtracting L-proline from the total amino acid pool. Aromatic precursor nitrogen was calculated as the sum of the nitrogen contributions of the quantified aromatic amino acids, including L-aspartic acid, L-threonine, L-valine, L-phenylalanine, L-leucine, L-isoleucine and L-tyrosine when detected.

### 4.7. Statistical Analysis

The statistical analysis of the studied parameters in vines and berries was performed using a variance analysis (one-way ANOVA) by Statgraphics Centurion XVI.I. The normality of residuals and homogeneity of variance were assessed prior to ANOVA using the Shapiro–Wilk and Levene tests, respectively. The differences between samples were compared using the Duncan test at 95% probability level. Pearson correlation analysis was performed to evaluate the relationships between the main groups of variables, including microclimatic and bioclimatic parameters, vine physiological parameters, yield components, berry maturity, bunch sunburn, sanitary status, and berry amino acid composition. This correlation analysis was performed using replicate-level data, with each experimental replicate considered as an independent observational unit. For each correlation coefficient, the associated *p*-value was calculated, and 95% confidence intervals were estimated to provide an indication of the precision of the correlation estimates. Principal component analysis (PCA) was performed using all data to classify treatments and parameters. PCA and Pearson analysis were performed using the Infostat 2014 software.

## 5. Conclusions

This single-season field study provides preliminary evidence that conventional and photoselective shading nets can modify cluster-zone radiation and influence bunch sunburn, powdery mildew severity, berry maturity, and specific amino acids in Cabernet Sauvignon under warm-climate vineyard conditions. The results suggest that the effects of shading nets on Cabernet Sauvignon bunch condition were not explained by incident PAR reduction alone. The conventional black Raschel net produced the strongest attenuation of incident PAR, but this reduction was not associated with lower sunburn or powdery mildew severity. In contrast, the gray–pearl photoselective net maintained intermediate radiation levels and showed lower sunburn and powdery mildew severity, suggesting that the observed responses may not be fully explained by nominal shading percentage alone. Future multi-season and multi-site studies, including different cultivars, canopy architectures, and direct measurements of berry surface temperature and other bunch-zone microclimatic variables, are needed before broader recommendations can be made.

## Figures and Tables

**Table 1:** Cluster-zone incident radiation, meteorological, bioclimatic, and thermal risk indices measured in Cabernet Sauvignon vines: control (uncovered), basal leaf removal, conventional (Raschel: 35% of shading) and photoselective shading nets (black–white and gray–pearl nets: 16% and 22% of shading, respectively).

	Control	Leaf Removal	Raschel	Black–White	Gray–Pearl
Incident radiation					
Photosynthetically active radiation * (PAR)	707.0 ± 9.9 c	844.0 ± 29.7 d	323.5 ± 84.1 a	537.0 ± 46.2 b	454.0 ± 99.0 b
Meteorological indices					
Minimum temperature (°C)	10.37 ± 0.21 a	10.02 ± 0.40 a	10.10 ± 0.45 a	10.21 ± 0.84 a	10.54 ± 0.58 a
Maximum temperature (°C)	34.60 ± 0.40 a	35.21 ± 0.01 a	33.54 ± 1.00 a	36.17 ± 1.22 a	34.64 ± 1.71 a
Average temperature (°C)	21.26 ± 0.56 a	20.85 ± 0.23 a	20.59 ± 0.46 a	20.71 ± 0.03 a	21.30 ± 0.49 a
Bioclimatic indices					
Growing degree days (GDD)	705.91 ± 123.30 a	797.01 ± 8.09 a	772.71 ± 33.57 a	782.67 ± 0.55 a	820.01 ± 29.95 a
Cool night index (CI)	10.02 ± 0.14 a	9.63 ± 0.23 a	9.90 ± 0.46 a	9.97 ± 0.95 a	10.21 ± 0.76 a
Risk indices					
N° days T° > 35 at afternoon	38.19 ± 6.81 a	36.5 ± 0.7 a	23.0 ± 8.5 a	44.0 ± 8.5 a	30.0 ± 15.6 a
Number of hours > 30 °C	297.00 ± 36.77 a	327.5 ± 34.7 a	282.5 ± 24.8 a	348.5 ± 31.8 a	333.5 ± 43.1 a
Number of hours > 35	72 ± 44 a	110 ± 23 a	56 ± 25 a	150 ± 52 a	97 ± 74 a

* PAR (µmol m^−2^ s^−1^). Mean values are shown with their corresponding standard deviation (*n* = 3). Different letters within each row indicate significant differences between treatments according to Duncan’s multiple range test (*p*-value ≤ 0.05).

**Table 2:** Yield components, berry maturity parameters, bunch sunburn, and powdery mildew status at harvest in Cabernet Sauvignon vines: control (uncovered), basal leaf removal, conventional (Raschel: 35% of shading) and photoselective shading nets (black–white and gray–pearl nets: 16% and 22% of shading, respectively).

	Control	Leaf Removal	Raschel	Black–White	Gray–Pearl
Yield components					
Yield per plant (kg)	2.49 ± 0.55 a	2.89 ± 1.12 a	2.66 ± 1.07 a	2.22 ± 0.65 a	2.59 ± 0.90 a
Number of bunches	24.89 ± 5.37 a	34.11 ± 9.16 b	29.00 ± 10.82 ab	23.00 ± 5.04 a	28.56 ± 5.17 ab
Bunch weight (kg)	0.10 ± 0.02 a	0.08 ± 0.02 a	0.09 ± 0.02 a	0.09 ± 0.02 a	0.09 ± 0.02 a
Berry parameters					
Berry weight (g) *	58.77 ± 9.67 a	66.02 ± 7.56 a	77.50 ± 29.40 a	60.47 ± 3.90 a	61.74 ± 8.72 a
Soluble solids (°Brix)	23.73 ± 0.55 b	22.43 ± 0.64 b	23.03 ± 1.44 b	20.55 ± 0.07 a	23.85 ± 0.49 b
pH	2.92 ± 0.02 b	2.82 ± 0.05 b	2.70 ± 0.03 a	2.83 ± 0.09 b	3.12 ± 0.04 c
Bunch sunburn					
Sunburn severity (%)	12.85 ± 3.83 ab	19.06 ± 5.26 bc	20.14 ± 7.36 c	16.04 ± 7.86 abc	11.63 ± 4.66 a
Sunburn incidence (%)	76.70 ± 15.79 a	86.47 ± 9.15 a	79.27 ± 21.96 a	79.01 ± 10.12 a	77.52 ± 20.10 a
Sanitary status					
Powdery mildew severity (%)	18.71 ± 7.98 bc	11.74 ± 4.59 ab	20.96 ± 8.80 c	12.25 ± 6.04 ab	7.28 ± 2.73 a
Powdery mildew incidence (%)	63.55 ± 30.48 a	75.18 ± 20.07 ab	86.81 ± 14.08 b	74.95 ± 19.02 ab	62.63 ± 13.78 a

* Berry weight was expressed as the mean of fifty berries in grams. Mean values are shown with their corresponding standard deviation (*n* = 3). Different letters within each row indicate significant differences between treatments according to Duncan’s multiple range test (*p*-value ≤ 0.05).

**Table 3:** Individual amino acid content, total amino acids, proline-to-arginine ratio, and aromatic precursor nitrogen in Cabernet Sauvignon berries: control (uncovered), basal leaf removal, conventional (Raschel: 35% of shading) and photoselective shading nets (black–white and gray–pearl nets: 16% and 22% of shading, respectively).

	Control	Leaf Removal	Raschel	Black–White	Gray–Pearl
L-Arginine	2.24 ± 1.48 ab	3.68 ± 0.60 b	3.48 ± 0.30 b	3.43 ± 0.09 b	1.40 ± 0.01 a
L-Glutamine	6.87 ± 0.36 a	7.68 ± 3.62 a	8.16 ± 2.05 a	5.42 ± 1.83 a	6.66 ± 0.49 a
L-Serine	1.25 ± 0.32 a	1.30 ± 0.21 a	1.37 ± 0.14 a	1.24 ± 0.23 a	1.42 ± 0.11 a
L-Aspartic acid	0.24 ± 0.04 a	0.33 ± 0.01 a	0.25 ± 0.06 a	0.30 ± 0.05 a	0.34 ± 0.00 a
L-Glutamic acid	1.13 ± 0.15 a	1.00 ± 0.24 a	1.05 ± 0.17 a	0.96 ± 0.37 a	1.21 ± 0.19 a
L-Threonine	2.07 ± 0.52 a	2.19 ± 0.25 a	1.91 ± 0.23 a	1.86 ± 0.39 a	2.18 ± 0.15 a
L-Glycine	8.32 ± 1.95 a	7.60 ± 0.37 a	7.04 ± 1.33 a	6.72 ± 0.89 a	7.70 ± 1.28 a
L-Alanine	6.95 ± 1.96 a	6.28 ± 0.67 a	5.67 ± 0.84 a	5.21 ± 1.03 a	6.32 ± 1.05 a
L-Proline	32.64 ± 8.20 a	35.84 ± 3.87 a	29.32 ± 4.76 a	27.79 ± 6.14 a	35.27 ± 7.57 a
L-Histidine	2.91 ± 0.97 ab	1.62 ± 0.14 a	4.56 ± 0.90 b	2.91 ± 0.89 ab	2.78 ± 0.61 ab
Proline-to-Arginine Ratio	22.12 ± 14.34 a	18.00 ± 11.09 a	8.53 ± 2.11 a	8.10 ± 1.75 a	25.61 ± 7.37 a
Total Amino Acids	57.53 ± 14.76 a	67.20 ± 8.54 a	58.31 ± 8.96 a	53.62 ± 11.47 a	71.29 ± 5.67 a
Total Amino Acids—Pro	32.66 ± 0.41 a	27.96 ± 6.75 a	28.99 ± 4.20 a	25.84 ± 5.33 a	36.02 ± 7.92 a
Aromatic Precursor N	2.71 ± 0.17 a	2.18 ± 0.61 a	2.10 ± 0.36 a	2.16 ± 0.39 a	2.43 ± 0.17 a

Pro: proline. N: nitrogen. Amino acids, total amino acids, total amino acids excluding proline, and aromatic precursor nitrogen are expressed as mg N kg^−1^. Mean values are shown with their corresponding standard deviation (*n* = 3). Different letters within each row indicate significant differences between treatments according to Duncan’s multiple range test (*p*-value ≤ 0.05).

## Data Availability

The original contributions presented in this study are included in the article. Further inquiries can be directed to the corresponding author.

## Supplementary Materials

The following supporting information can be downloaded at https://www.mdpi.com/article/10.3390/plants15142183/s1. Figure S1: Exploratory Pearson correlation heatmaps of measured variables in Cabernet Sauvignon vines subjected to shading-net treatments. (Top) Agronomic, physiological, thermal, radiation, bunch sunburn, and powdery mildew variables. (Bottom) Free amino acids, derived nitrogen indices, and selected agronomic and microclimatic variables. Figure S2: Aerial view of the experimental treatment layout on the day of shading-net installation. The images show the spatial arrangement of the vineyard rows and the distribution of the treatment plots used for the evaluation of conventional and photoselective shading nets in Cabernet Sauvignon.
