# Supplementary material for: Shading Nets Modified Cluster-Zone Radiation, Bunch Sunburn Percentage and Berry Amino Acid Content in Cabernet Sauvignon: A Preliminary Study
Source: Plants (Basel). 2026 Jul 16;15(14):2183. doi: 10.3390/plants15142183 (PMC13414722; doi:10.3390/plants15142183)
Supplement: Supplementary file 1 [file plants-15-02183-s001.zip › plants-4412691-supplementary.pdf]

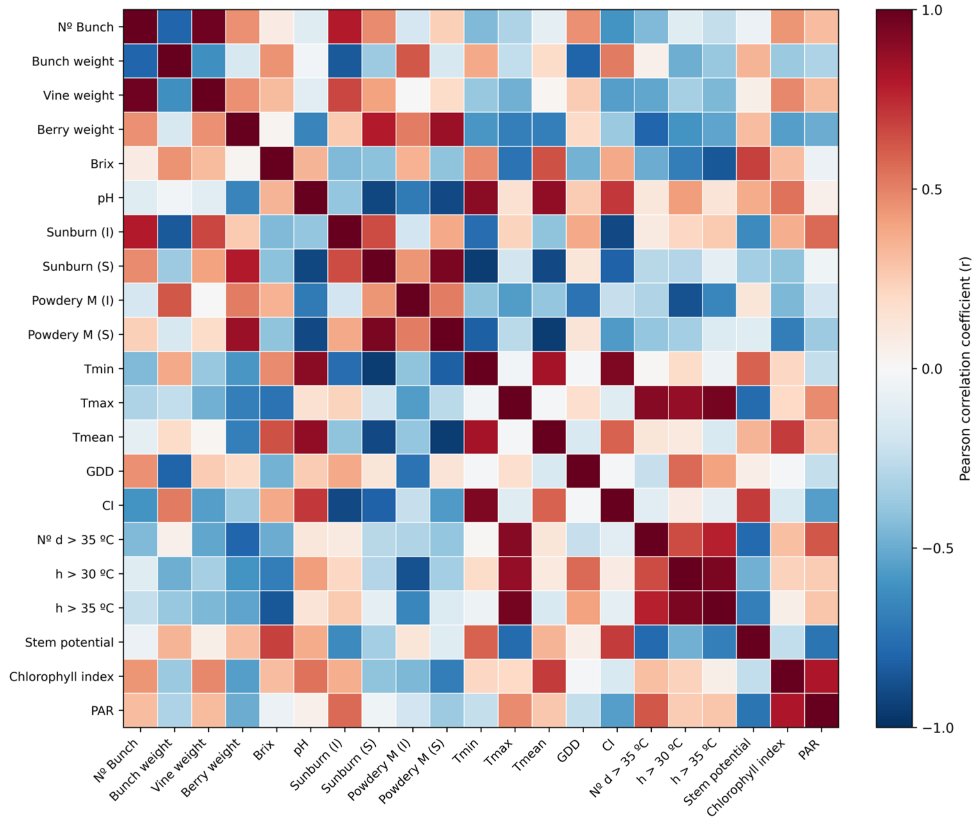

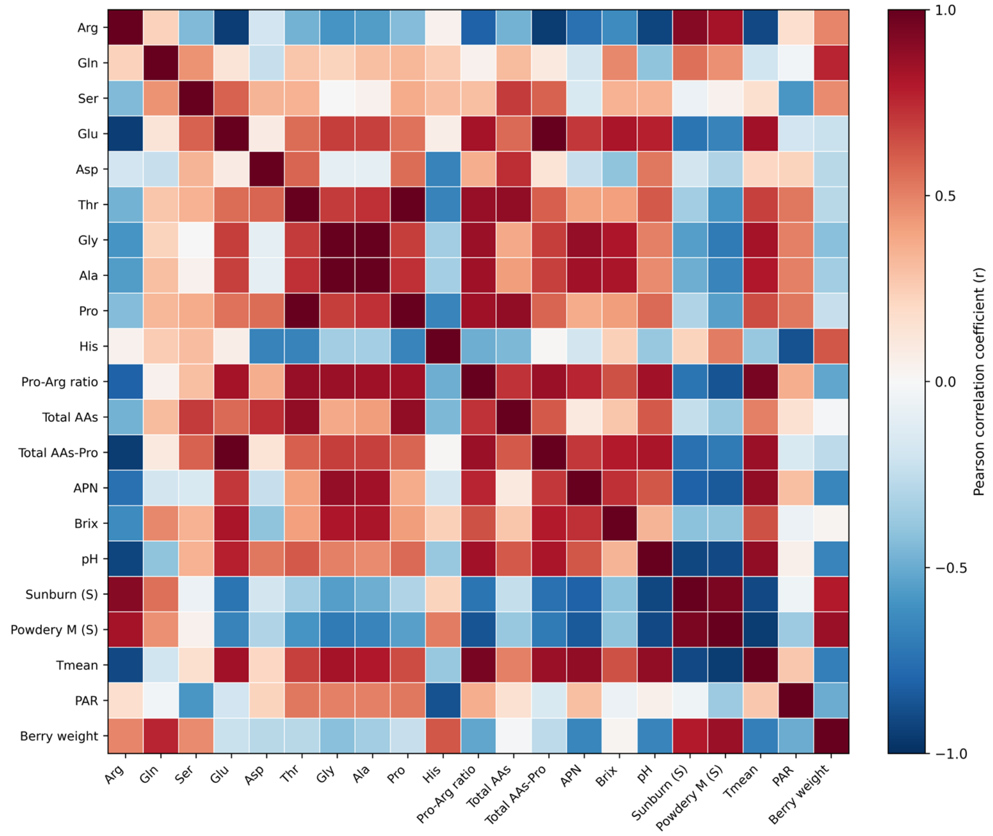

Supplementary Figure 1. Exploratory Pearson correlation heatmaps of measured variables in Cabernet Sauvignon vines subjected to shading-net treatments. (upper) Agronomic, physiological, thermal, radiation, bunch sunburn, and powdery mildew variables. (bottom) Free amino acids, derived nitrogen indices, and selected agronomic and microclimatic variables.

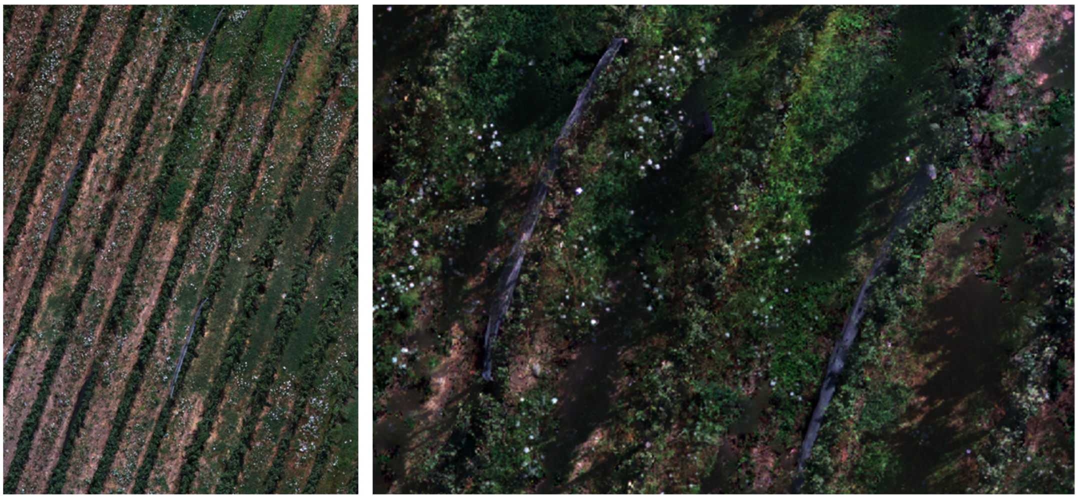

Supplementary Figure 2. Aerial view of the experimental treatment layout on the day of shading-net installation. The images show the spatial arrangement of the vineyard rows and the distribution of the treatment plots used for the evaluation of conventional and photoselective shading nets in Cabernet Sauvignon.
